# 2-{4-[Bis(4-bromo­phen­yl)amino]­benzyl­idene}malono­nitrile

**DOI:** 10.1107/S1600536814013816

**Published:** 2014-06-21

**Authors:** Yu-Jian Zhang, Sheng-Liang Ni

**Affiliations:** a Department of Material Chemistry, Huzhou University, Huzhou, Zhejiang 313000, People’s Republic of China

**Keywords:** crystal structure

## Abstract

In the crystal structure of the title compound, C_22_H_13_Br_2_N_3_, the two bromo­phenyl rings are rotated out of the plane of the central benzyl­idene ring by 68.7 (1) and 69.3 (1)°. Both cyano substituents are located nearly in the plane of the benzylidene ring, with the mean plane of the methylmalononitrile group being inclined to this ring by 5.8 (1)°. In the crystal, the mol­ecules are linked by weak C—H⋯N hydrogen bonds into layers parallel to the *bc* plane.

## Related literature   

For general background to aryl­amines such as tri­phenyl­amine as versatile optical materials, see: Ning *et al.* (2007 ▶); Noh *et al.* (2010 ▶). For related luminescent and electron-donating materials, see: Yao & Belfield (2005 ▶); Patra *et al.* (2007 ▶); Zhang *et al.* (2012 ▶). For the synthesis of the title compound, see: Chiang *et al.* (2005 ▶). 
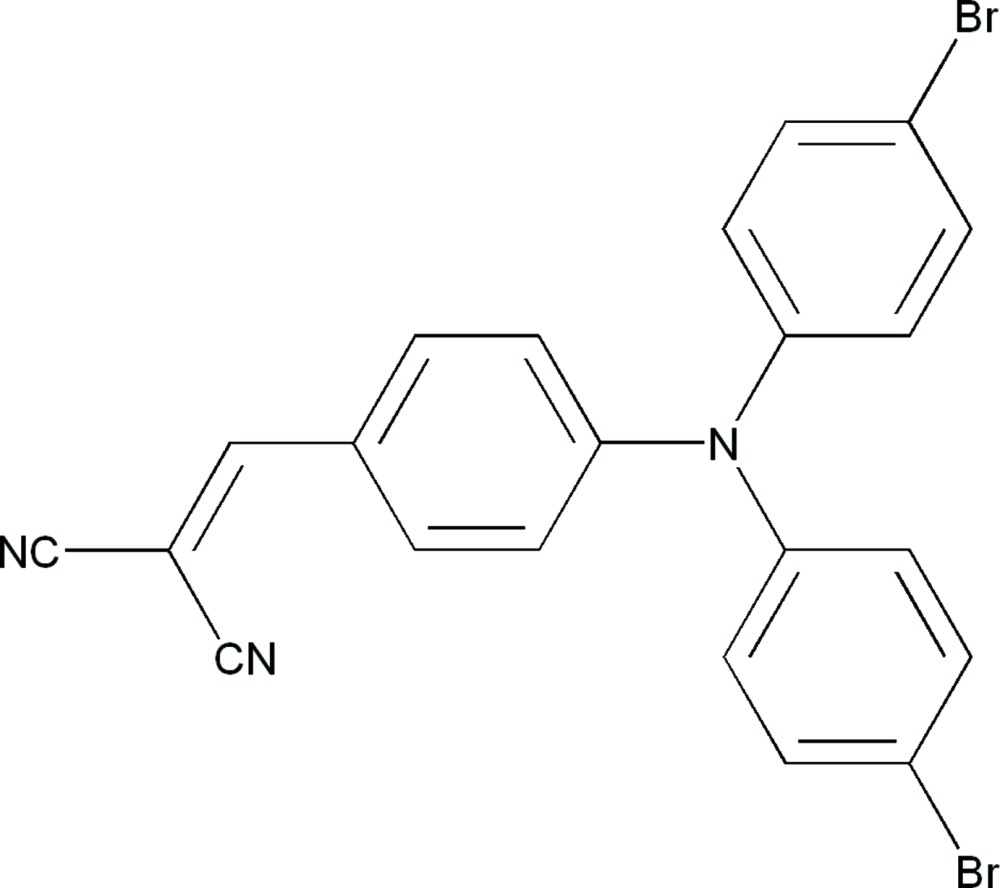


## Experimental   

### 

#### Crystal data  

C_22_H_13_Br_2_N_3_
*M* *_r_* = 479.17
Monoclinic, 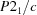

*a* = 14.6846 (6) Å
*b* = 10.3997 (4) Å
*c* = 13.1961 (4) Å
β = 103.501 (4)°
*V* = 1959.56 (12) Å^3^
*Z* = 4
Mo *K*α radiation
μ = 4.15 mm^−1^
*T* = 298 K
0.30 × 0.20 × 0.20 mm

#### Data collection  

Oxford Diffraction CrysAlis CCD diffractometer
Absorption correction: multi-scan (*CrysAlis RED*; Oxford Diffraction, 2006 ▶) *T*_min_ = 0.369, *T*_max_ = 0.491
9075 measured reflections
3843 independent reflections
2702 reflections with *I* > 2σ(*I*)
*R*_int_ = 0.024

#### Refinement  

*R*[*F* ^2^ > 2σ(*F* ^2^)] = 0.041
*wR*(*F* ^2^) = 0.094
*S* = 1.02
3843 reflections
244 parameters
H-atom parameters constrained
Δρ_max_ = 0.38 e Å^−3^
Δρ_min_ = −0.57 e Å^−3^

### 

Data collection: *CrysAlis CCD* (Oxford Diffraction, 2006 ▶); cell refinement: *CrysAlis CCD*; data reduction: *CrysAlis RED* (Oxford Diffraction, 2006 ▶); program(s) used to solve structure: *SHELXS97* (Sheldrick, 2008 ▶); program(s) used to refine structure: *SHELXL97* (Sheldrick, 2008 ▶); molecular graphics: *SHELXTL* (Sheldrick, 2008 ▶); software used to prepare material for publication: *SHELXL97*.

## Supplementary Material

Crystal structure: contains datablock(s) ItcLa, I. DOI: 10.1107/S1600536814013816/nc2326sup1.cif

Structure factors: contains datablock(s) I. DOI: 10.1107/S1600536814013816/nc2326Isup2.hkl

Supporting information file. DOI: 10.1107/S1600536814013816/nc2326Isup3.cml

CCDC reference: 1008127

Additional supporting information:  crystallographic information; 3D view; checkCIF report

## Figures and Tables

**Table 1:** Hydrogen-bond geometry (Å, °)

*D*—H⋯*A*	*D*—H	H⋯*A*	*D*⋯*A*	*D*—H⋯*A*
C4—H4*A*⋯N3^i^	0.93	2.52	3.438 (5)	169
C6—H6*A*⋯N2^ii^	0.93	2.60	3.404 (5)	145

Symmetry codes: (i) 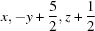; (ii) 
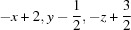
.

**Fig. 1.**
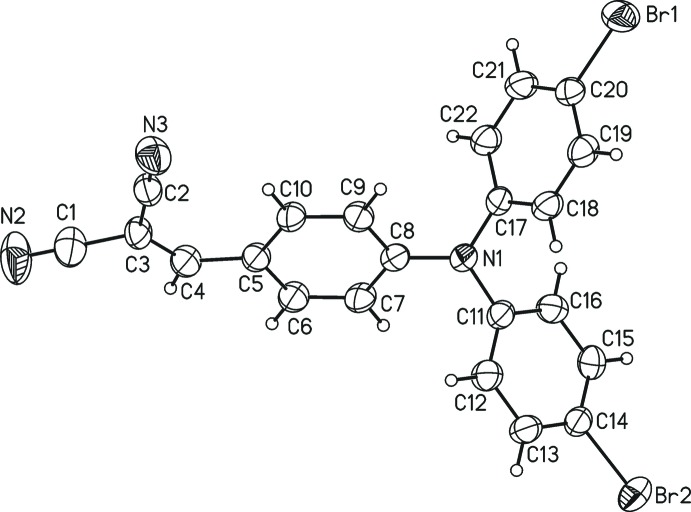
ORTEP view of the title compound with labeling and displacement ellipsoids drawn at 45% probability level.

## supplementary crystallographic information

### S1. Comment

Arylamines such as triphenylamine (TPA) are versatile optical materials. The TPA derivatives containing the twisty arylamine exhibits good electron-donating ability and restrict the formation of an excimer complex, which further contributes to enhance fluorescence quantum efficiency (Ning *et al.*, 2007; Noh *et al.*, 2010). As a result, the fluorescence molecule with TPA are the excellent candidate as the luminescent and electron-donating materials (Yao & Belfield, 2005; Patra *et al.*, 2007). Moreover, the molecular packing is relatively loose, leading to the piezofluorochromic properties due to the twisted conformation in the aggregated state. Our group always investigated the piezofluorochromic and optical properties of triphenylamine (TPA) derivatives (Zhang *et al.*, 2012). It was found that the tiny change of the molecular structure had great effect on the piezofluorochromic behavior. The, dye DiCN-TPA with a simple molecular structure is an excellent candidate to investigate structure- property relationships. Within this project the crstal structure of the title compound was determined.

The asymmetric unit consists of one molecule in a general position (Fig. 1). In the crystal structure,the two bromophenyl groups are rotated out of the central benzylidene ring by 68.7 (1) ° and 69.3 (1) °. Both cyano substituents are located nearly in the plane of the 6-membered ring and the dihedral angle between the plane C5–C10 and N2, N3 and C1 to C4 amount to 5.8 (1) °. In the crystal, the molecules self–assemble to form a hydrogen–bonded layer parallel to the *bc* crystallographic plane connected by weak C—H···N hydrogen bonds(C4—H4A···N3^#1^ = 169 °, C4···N3^#1^ = 3.438 (5) Å, C6—H6A···N2^#2^= 145 ° and C6···N2^#2^ = 3.404 (5) Å) (#1 = *x*, 5/2 - *y*, 1/2 + *z*; #2 = 2 - *x*, -1/2 + *y*, 3/2 - *z*). These layers are stacked along *a* axis and are stabilized by van der Waals interactions.

### S2. Experimental

The title compound was prepared by the reaction of 4–(bis(4–bromophenyl)amino) benzaldehyde (DiBr–TPA) and malononitrile (Chiang *et al.*,2005) DiBr–TPA (1.3 g, 3 mmol), malononitrile (0.8 g, 12.1 mmol), and basic aluminium oxide (Al_2_O_3_, 4.0 g) are stirred in toluene (30 ml) for 18 h at 100 °C. And then, the hot solution was cooled down to room temperature, the reaction solution was filtered. The crude product was purified by column chromatography using CH_2_Cl_2_/hexane (1/50) mixture as eluent to obtain the compound (silica gel, ethyl acetate/petroleum ether: 1/9). A yellow powders was obtained with the yield of 43.1%. The yellow needle-like crystal was obtained by slowly evaporating a solution of DiCN–TPA in the mixed solvent (CH_2_Cl_2_/hexane = 1/9, *v*/*v*) at room temperature for two weeks.

### S3. Refinement

All C—H H-atoms bonded were positioned with idealized geometry and refined isotropic with *U*_iso_(H) = 1.2 *U*_eq_(C) using a riding model.

### Crystal data

**Table:**

C_22_H_13_Br_2_N_3_	*F*(000) = 944
*M**_r_* = 479.17	*D*_x_ = 1.624 Mg m^−^^3^
Monoclinic, *P*2_1_/*c*	Mo *K*α radiation, λ = 0.71073 Å
Hall symbol: -P 2ybc	Cell parameters from 2681 reflections
*a* = 14.6846 (6) Å	θ = 2.9–29.3°
*b* = 10.3997 (4) Å	µ = 4.15 mm^−^^1^
*c* = 13.1961 (4) Å	*T* = 298 K
β = 103.501 (4)°	Needle-like, yellow
*V* = 1959.56 (12) Å^3^	0.30 × 0.20 × 0.20 mm
*Z* = 4	

### Data collection

**Table:**

Oxford Diffraction CrysAlis CCD diffractometer	3843 independent reflections
Radiation source: Enhance (Mo) X-ray Source	2702 reflections with *I* > 2σ(*I*)
Graphite monochromator	*R*_int_ = 0.024
ω scans	θ_max_ = 26.0°, θ_min_ = 3.2°
Absorption correction: multi-scan (*CrysAlis RED*; Oxford Diffraction, 2006)	*h* = −18→18
*T*_min_ = 0.369, *T*_max_ = 0.491	*k* = −7→12
9075 measured reflections	*l* = −14→16

### Refinement

**Table:**

Refinement on *F*^2^	Primary atom site location: structure-invariant direct methods
Least-squares matrix: full	Secondary atom site location: difference Fourier map
*R*[*F*^2^ > 2σ(*F*^2^)] = 0.041	Hydrogen site location: inferred from neighbouring sites
*wR*(*F*^2^) = 0.094	H-atom parameters constrained
*S* = 1.02	*w* = 1/[σ^2^(*F*_o_^2^) + (0.0359*P*)^2^ + 1.3631*P*] where *P* = (*F*_o_^2^ + 2*F*_c_^2^)/3
3843 reflections	(Δ/σ)_max_ = 0.003
244 parameters	Δρ_max_ = 0.38 e Å^−^^3^
0 restraints	Δρ_min_ = −0.57 e Å^−^^3^

### Special details

**Table:**

Experimental. Absorption correction: *CrysAlis RED*, Oxford Diffraction Ltd., Version 1.171.32.5 (release 08–05-2007 CrysAlis171. NET) (compiled May 8 2007,13:10:02) Empirical absorption correction using spherical harmonics, implemented in SCALE3 ABSPACK scaling algorithm.
Geometry. All e.s.d.'s (except the e.s.d. in the dihedral angle between two l.s. planes) are estimated using the full covariance matrix. The cell e.s.d.'s are taken into account individually in the estimation of e.s.d.'s in distances, angles and torsion angles; correlations between e.s.d.'s in cell parameters are only used when they are defined by crystal symmetry. An approximate (isotropic) treatment of cell e.s.d.'s is used for estimating e.s.d.'s involving l.s. planes.
Refinement. Refinement of *F*^2^ against ALL reflections. The weighted *R*-factor *wR* and goodness of fit *S* are based on *F*^2^, conventional *R*-factors *R* are based on *F*, with *F* set to zero for negative *F*^2^. The threshold expression of *F*^2^ > σ(*F*^2^) is used only for calculating *R*-factors(gt) *etc*. and is not relevant to the choice of reflections for refinement. *R*-factors based on *F*^2^ are statistically about twice as large as those based on *F*, and *R*- factors based on ALL data will be even larger.

### Fractional atomic coordinates and isotropic or equivalent isotropic displacement parameters (Å^2^)

**Table:**

	*x*	*y*	*z*	*U*_iso_*/*U*_eq_	
N1	0.7072 (2)	0.7143 (3)	0.4629 (2)	0.0468 (7)	
N2	1.0781 (3)	1.4223 (5)	0.6211 (3)	0.0959 (15)	
N3	0.9718 (2)	1.2359 (4)	0.3307 (3)	0.0669 (10)	
C1	1.0307 (3)	1.3420 (5)	0.5799 (3)	0.0624 (11)	
C2	0.9707 (2)	1.2363 (4)	0.4169 (3)	0.0470 (9)	
C3	0.9731 (3)	1.2405 (4)	0.5257 (3)	0.0451 (9)	
C4	0.9304 (3)	1.1577 (4)	0.5774 (3)	0.0468 (9)	
H4A	0.9400	1.1749	0.6483	0.056*	
C5	0.8725 (2)	1.0472 (3)	0.5432 (2)	0.0414 (8)	
C6	0.8430 (2)	0.9732 (4)	0.6183 (2)	0.0469 (9)	
H6A	0.8602	0.9987	0.6877	0.056*	
C7	0.7902 (3)	0.8654 (4)	0.5933 (2)	0.0458 (9)	
H7A	0.7731	0.8181	0.6458	0.055*	
C8	0.7612 (2)	0.8249 (3)	0.4900 (2)	0.0407 (8)	
C9	0.7887 (3)	0.8989 (4)	0.4134 (2)	0.0473 (9)	
H9A	0.7700	0.8746	0.3438	0.057*	
C10	0.8428 (2)	1.0064 (4)	0.4398 (2)	0.0461 (9)	
H10A	0.8602	1.0536	0.3875	0.055*	
C11	0.7119 (2)	0.6094 (3)	0.5341 (2)	0.0413 (8)	
C12	0.7968 (2)	0.5562 (4)	0.5836 (3)	0.0501 (9)	
H12A	0.8522	0.5923	0.5742	0.060*	
C13	0.8002 (2)	0.4497 (4)	0.6470 (3)	0.0480 (9)	
H13A	0.8574	0.4137	0.6802	0.058*	
C14	0.7180 (2)	0.3981 (3)	0.6603 (2)	0.0426 (8)	
C15	0.6331 (2)	0.4516 (4)	0.6153 (3)	0.0496 (9)	
H15A	0.5781	0.4168	0.6269	0.060*	
Br1	0.43314 (3)	0.65644 (5)	0.04171 (3)	0.06879 (17)	
C16	0.6305 (2)	0.5586 (4)	0.5520 (3)	0.0484 (9)	
H16A	0.5733	0.5964	0.5214	0.058*	
C17	0.6453 (2)	0.7022 (3)	0.3628 (2)	0.0400 (8)	
C18	0.6473 (3)	0.5930 (4)	0.3033 (3)	0.0477 (9)	
H18A	0.6907	0.5285	0.3279	0.057*	
C19	0.5850 (3)	0.5793 (4)	0.2072 (3)	0.0502 (9)	
H19A	0.5861	0.5056	0.1676	0.060*	
C20	0.5219 (2)	0.6753 (3)	0.1711 (2)	0.0440 (9)	
C21	0.5201 (3)	0.7855 (4)	0.2277 (3)	0.0491 (9)	
H21A	0.4777	0.8507	0.2018	0.059*	
C22	0.5820 (3)	0.7985 (4)	0.3238 (3)	0.0482 (9)	
H22A	0.5810	0.8730	0.3625	0.058*	
Br2	0.72147 (3)	0.24595 (4)	0.74116 (4)	0.07151 (17)	

### Atomic displacement parameters (Å^2^)

**Table:**

	*U*^11^	*U*^22^	*U*^33^	*U*^12^	*U*^13^	*U*^23^
N1	0.0552 (18)	0.0382 (18)	0.0415 (15)	−0.0087 (16)	0.0003 (13)	0.0082 (13)
N2	0.141 (4)	0.093 (3)	0.055 (2)	−0.065 (3)	0.026 (2)	−0.022 (2)
N3	0.073 (2)	0.080 (3)	0.047 (2)	−0.018 (2)	0.0127 (17)	−0.0022 (18)
C1	0.087 (3)	0.063 (3)	0.040 (2)	−0.022 (3)	0.021 (2)	−0.007 (2)
C2	0.049 (2)	0.047 (2)	0.044 (2)	−0.0095 (19)	0.0077 (16)	−0.0032 (17)
C3	0.057 (2)	0.040 (2)	0.0378 (18)	−0.0081 (19)	0.0088 (16)	−0.0075 (16)
C4	0.061 (2)	0.045 (2)	0.0330 (16)	−0.003 (2)	0.0094 (16)	−0.0063 (16)
C5	0.052 (2)	0.038 (2)	0.0326 (16)	−0.0026 (18)	0.0070 (15)	−0.0015 (15)
C6	0.063 (2)	0.044 (2)	0.0316 (16)	−0.004 (2)	0.0079 (16)	−0.0021 (16)
C7	0.061 (2)	0.045 (2)	0.0321 (16)	−0.004 (2)	0.0119 (16)	0.0054 (16)
C8	0.0444 (19)	0.036 (2)	0.0405 (17)	0.0017 (17)	0.0075 (15)	0.0037 (15)
C9	0.062 (2)	0.046 (2)	0.0310 (17)	−0.007 (2)	0.0053 (16)	−0.0011 (16)
C10	0.062 (2)	0.041 (2)	0.0337 (16)	−0.008 (2)	0.0066 (16)	0.0035 (15)
C11	0.051 (2)	0.0346 (19)	0.0367 (17)	−0.0033 (18)	0.0079 (15)	0.0022 (15)
C12	0.040 (2)	0.053 (3)	0.057 (2)	−0.0042 (19)	0.0122 (17)	0.0134 (19)
C13	0.043 (2)	0.046 (2)	0.054 (2)	0.0045 (19)	0.0104 (17)	0.0130 (18)
C14	0.052 (2)	0.035 (2)	0.0400 (18)	−0.0036 (18)	0.0094 (16)	0.0011 (15)
C15	0.044 (2)	0.049 (2)	0.057 (2)	−0.012 (2)	0.0133 (17)	0.0034 (19)
Br1	0.0867 (3)	0.0633 (3)	0.0449 (2)	0.0030 (3)	−0.00761 (19)	−0.0020 (2)
C16	0.041 (2)	0.051 (2)	0.050 (2)	0.0016 (19)	0.0056 (16)	0.0080 (18)
C17	0.046 (2)	0.0343 (18)	0.0387 (17)	−0.0067 (18)	0.0072 (15)	0.0031 (15)
C18	0.056 (2)	0.035 (2)	0.049 (2)	0.0065 (19)	0.0071 (17)	0.0022 (17)
C19	0.069 (2)	0.038 (2)	0.0426 (19)	0.001 (2)	0.0118 (18)	−0.0046 (17)
C20	0.052 (2)	0.041 (2)	0.0370 (17)	−0.0025 (19)	0.0066 (15)	0.0047 (16)
C21	0.055 (2)	0.040 (2)	0.049 (2)	0.0092 (19)	0.0045 (17)	0.0026 (17)
C22	0.059 (2)	0.036 (2)	0.0460 (19)	0.004 (2)	0.0064 (17)	−0.0016 (17)
Br2	0.0765 (3)	0.0511 (3)	0.0828 (3)	−0.0111 (2)	0.0102 (2)	0.0252 (2)

### Geometric parameters (Å, º)

**Table:**

N1—C8	1.395 (4)	C11—C12	1.381 (5)
N1—C17	1.424 (4)	C12—C13	1.382 (5)
N1—C11	1.431 (4)	C12—H12A	0.9300
N2—C1	1.140 (5)	C13—C14	1.369 (5)
N3—C2	1.142 (4)	C13—H13A	0.9300
C1—C3	1.436 (6)	C14—C15	1.368 (5)
C2—C3	1.428 (5)	C14—Br2	1.903 (3)
C3—C4	1.341 (5)	C15—C16	1.386 (5)
C4—C5	1.438 (5)	C15—H15A	0.9300
C4—H4A	0.9300	Br1—C20	1.900 (3)
C5—C10	1.399 (4)	C16—H16A	0.9300
C5—C6	1.401 (5)	C17—C22	1.382 (5)
C6—C7	1.359 (5)	C17—C18	1.384 (5)
C6—H6A	0.9300	C18—C19	1.387 (5)
C7—C8	1.396 (4)	C18—H18A	0.9300
C7—H7A	0.9300	C19—C20	1.370 (5)
C8—C9	1.403 (5)	C19—H19A	0.9300
C9—C10	1.368 (5)	C20—C21	1.372 (5)
C9—H9A	0.9300	C21—C22	1.384 (5)
C10—H10A	0.9300	C21—H21A	0.9300
C11—C16	1.377 (5)	C22—H22A	0.9300
			
C8—N1—C17	120.8 (3)	C11—C12—H12A	119.7
C8—N1—C11	121.5 (3)	C13—C12—H12A	119.7
C17—N1—C11	117.6 (3)	C14—C13—C12	118.8 (3)
N2—C1—C3	178.0 (5)	C14—C13—H13A	120.6
N3—C2—C3	177.4 (4)	C12—C13—H13A	120.6
C4—C3—C2	126.0 (3)	C15—C14—C13	121.8 (3)
C4—C3—C1	120.6 (3)	C15—C14—Br2	118.9 (3)
C2—C3—C1	113.3 (3)	C13—C14—Br2	119.2 (3)
C3—C4—C5	131.7 (3)	C14—C15—C16	118.8 (3)
C3—C4—H4A	114.2	C14—C15—H15A	120.6
C5—C4—H4A	114.2	C16—C15—H15A	120.6
C10—C5—C6	116.4 (3)	C11—C16—C15	120.6 (3)
C10—C5—C4	125.2 (3)	C11—C16—H16A	119.7
C6—C5—C4	118.4 (3)	C15—C16—H16A	119.7
C7—C6—C5	122.4 (3)	C22—C17—C18	119.0 (3)
C7—C6—H6A	118.8	C22—C17—N1	120.7 (3)
C5—C6—H6A	118.8	C18—C17—N1	120.3 (3)
C6—C7—C8	120.8 (3)	C17—C18—C19	120.4 (3)
C6—C7—H7A	119.6	C17—C18—H18A	119.8
C8—C7—H7A	119.6	C19—C18—H18A	119.8
N1—C8—C7	121.6 (3)	C20—C19—C18	119.5 (3)
N1—C8—C9	120.6 (3)	C20—C19—H19A	120.3
C7—C8—C9	117.7 (3)	C18—C19—H19A	120.3
C10—C9—C8	120.8 (3)	C19—C20—C21	121.1 (3)
C10—C9—H9A	119.6	C19—C20—Br1	120.3 (3)
C8—C9—H9A	119.6	C21—C20—Br1	118.6 (3)
C9—C10—C5	121.8 (3)	C20—C21—C22	119.2 (3)
C9—C10—H10A	119.1	C20—C21—H21A	120.4
C5—C10—H10A	119.1	C22—C21—H21A	120.4
C16—C11—C12	119.2 (3)	C17—C22—C21	120.8 (3)
C16—C11—N1	119.7 (3)	C17—C22—H22A	119.6
C12—C11—N1	121.1 (3)	C21—C22—H22A	119.6
C11—C12—C13	120.6 (3)		

### Hydrogen-bond geometry (Å, º)

**Table:**

*D*—H···*A*	*D*—H	H···*A*	*D*···*A*	*D*—H···*A*
C4—H4*A*···N3^i^	0.93	2.52	3.438 (5)	169
C6—H6*A*···N2^ii^	0.93	2.60	3.404 (5)	145

Symmetry codes: (i) *x*, −*y*+5/2, *z*+1/2; (ii) −*x*+2, *y*−1/2, −*z*+3/2.
